# Odor of Plants

**Published:** 1873-01

**Authors:** 


THE ODOR OF PLANTS.—It may be laid down, as a general principle, that a larger proportion of white flowers are more fragrant than those of any other color; yellow come next, then red, and lastly blue; after which, and in the same order, may be reckond violet, green, orange, brown and black.

